# Conformational Response to Ligand Binding in Phosphomannomutase2: INSIGHTS INTO INBORN GLYCOSYLATION DISORDER*[Fn FN2]

**DOI:** 10.1074/jbc.M114.586362

**Published:** 2014-10-16

**Authors:** Giuseppina Andreotti, Israel Cabeza de Vaca, Angelita Poziello, Maria Chiara Monti, Victor Guallar, Maria Vittoria Cubellis

**Affiliations:** From the ‡ Istituto di Chimica Biomolecolare-Consiglio Nazionale Delle Ricerche, 80078 Pozzuoli, Italy,; § Joint Barcelona Supercomputing Center-Center for Genomic Regulation-Institute for Research in Biomedicine Research Program in Computational Biology, Barcelona Supercomputing Center, c/Jordi Girona 29, 08034 Barcelona, Spain,; ‖ Dipartimento di Farmacia, Università degli Studi di Salerno, 84084 Fisciano, Italy,; ** Institució Catalana de Recerca i Estudis Avançats, Passeig Lluís Companys 23, 08010 Barcelona, Spain, and; ¶ Dipartimento di Biologia, Università Federico II, 80126 Naples, Italy

**Keywords:** Computer Modeling, Drug Discovery, Glycosylation, Glycosylation Inhibitor, Ligand-binding Protein, 1,6-Bisphosphate, PELE, Phosphomannomutase

## Abstract

The most common glycosylation disorder is caused by mutations in the gene encoding phosphomannomutase2, producing a disease still without a cure. Phosphomannomutase2, a homodimer in which each chain is composed of two domains, requires a bisphosphate sugar (either mannose or glucose) as activator, opening a possible drug design path for therapeutic purposes. The crystal structure of human phosphomannomutase2, however, lacks bound substrate and a key active site loop. To speed up drug discovery, we present here the first structural model of a bisphosphate substrate bound to human phosphomannomutase2. Taking advantage of recent developments in all-atom simulation techniques in combination with limited and site-directed proteolysis, we demonstrated that α-glucose 1,6-bisphosphate can adopt two low energy orientations as required for catalysis. Upon ligand binding, the two domains come close, making the protein more compact, in analogy to the enzyme in the crystals from *Leishmania mexicana*. Moreover, proteolysis was also carried out on two common mutants, R141H and F119L. It was an unexpected finding that the mutant most frequently found in patients, R141H, although inactive, does bind α-glucose 1,6-bisphosphate and changes conformation.

## Teaser

**Background:** Mutations in phosphomannomutase2 cause glycosylation disorder, a disease without a cure that will largely benefit from accurate ligand-bound models.

**Results:** We obtained two models of phospomannomutase2 bound to glucose 1,6-bisphosphate and validated them with limited proteolysis.

**Conclusion:** Ligand binding induces a large conformational transition in PMM2.

**Significance:** We produce and validate closed-form models of PMM2 that represent a starting point for rational drug discovery.

## Introduction

Defects in human phosphomannomutase2 (PMM2)^4^ are the cause of an autosomal recessive glycosylation disorder, PMM2-CDG (OMIM entry 212065), also known as CDG1A or Jaeken syndrome. PMM2-CDG is characterized by underglycosylated glycoproteins and is associated with severe mental and psychomotor retardation (1). More than 85 missense mutations have been described for the gene encoding PMM2 (2), the most common involving an arginine in position 141, R141H (rs28936415; NM_000303.2: c.422G→A; NP_000294.1: p.R141H) with a frequency of 8 of 1000 in a default global population (1000 genome phase 1 genotype data from 1094 worldwide individuals), and 1 of 60/80 in Northern Europe populations (3). This mutation, which abolishes activity, has never been observed in homozygosity probably because complete lack of phosphomannomutase2 activity is lethal (4, 5). Often, patients are compound heterozygotes with at least one hypomorphic mutation (6). The second most frequent mutation, F119L (rs80338701; NM_000303.2: c.357C→A; NP_000294.1: p.F119L), which occurs at the interface between subunits and retains activity, has been observed in association with R141H but also in homozygous patients (5).

No pathologies have been associated yet to a paralogous enzyme, phosphomannomutase1 (PMM1), also present in humans (7). Both enzymes catalyze the reversible conversion of mannose 6-phosphate into mannose 1-phosphate or, to a smaller extent, the conversion of glucose 6-phosphate into glucose 1-phosphate (Glc-1-P), requiring activation by α-glucose 1,6-bisphosphate (Glc-1,6-P_2_) or α-mannose 1,6-bisphosphate (8). Twenty-one sequences and five crystal structures of phosphomannomutase from Eukaryota are known; the apo x-ray structure of PMM2 (Protein Data Bank code 2AMY) was solved as were those of PMM1 in the absence of sugar ligand (Protein Data Bank code 2FUC) and in the presence of mannose 1-phosphate (Protein Data Bank code 2FUE) (9). Phosphomannomutase from *Leishmania mexicana* (PMM_LEIME) (10) has been crystallized in the absence of sugar ligand (Protein Data Bank code 2I54) and in the presence of β-glucose 1,6-bisphosphate (Protein Data Bank code 2I55), and in this last case, the protein is seen in closed conformation. Moreover, only one chain is found in the asymmetric unit of 2AMY, 2FUE, and 2FUC, whereas 2I54 and 2I55 contain dimers; an equilibrium exists for PMM2 that is shifted toward the dimer in the wild type and toward the monomer in F119L (11).

Although many therapeutic strategies are being evaluated for PMM2-CDG (12–15), there is still no cure for this disease. Among others, enhancement of activators such as Glc-1,6-P_2_ above the normal concentration has been proposed (11). The screening for suitable drugs is time- and money-consuming but can certainly speed up rational design based on accurate structural protein-ligand knowledge. Regrettably, only the structure of the apoenzyme in the open conformation is available for PMM2. The comparison of PMM_LEIME structures 2I54 and 2I55 shows that it undergoes induced fit and a large domain rearrangement upon ligand binding. We hypothesize that a similar change occurs in PMM2. For this reason, before attempting structure-based virtual screening, a suitable model of the receptor enzyme in closed form has to be built.

In this study, our hypothesis was analyzed by combining experimental and computational techniques. All-atom computational modeling has experienced a remarkable step forward with recent developments in software and hardware. Accurate non-biased ligand migration and binding associated to microsecond time scale protein dynamics are readily obtained now by means of molecular dynamics (16) or Monte Carlo techniques (17). Using the Protein Energy Landscape Exploration (PELE) software (17, 18) based on a combination of a Monte Carlo technique with protein structure prediction algorithms, we demonstrate that PMM2 undergoes a large conformational change upon ligand binding that is similar to PMME_LEIME. Moreover, we produced two enzyme models in the presence of the activator Glc-1,6-P_2_ representing two alternative binding modes. To support the models, we carried out limited proteolysis on wild type PMM2 as well as on two common mutants, F119L and R141H. The latter inactive mutant binds the ligand and undergoes a conformational change, making it more resistant to proteases and thermal unfolding.

## EXPERIMENTAL PROCEDURES

DEAE-Sepharose Fast Flow, Superdex 75, and thrombin were purchased from GE Healthcare. Phosphoglucose isomerase from rabbit muscle, phosphomannose isomerase from *Escherichia coli*, glucose-6-phosphate dehydrogenase from bakers' yeast (*Saccharomyces cerevisiae*), α-d-glucose 1,6-bisphosphate potassium salt hydrate, α-d-glucose 1-phosphate disodium salt hydrate, and β-nicotinamide adenine dinucleotide phosphate sodium salt were purchased from Sigma-Aldrich. SYPRO Orange was from Invitrogen Molecular Probes. Sodium orthovanadate was from Acros Organics. All other reagents were of analytical grade.

### 

#### 

##### Protein Expression and Purification

Wild type, F119L, and R141H were expressed in *E. coli* BL21(DE3) strain grown at 37 °C in LB broth containing 0.2 mg/ml ampicillin. The expression and purification of wild type (WT) were performed as described (8) with only minor changes. The expression and purification of F119L were performed as described previously (11). The expression of R141H was assessed. The best production of the protein was obtained by adding 0.4 mm isopropyl 1-thio-β-d-galactopyranoside when the optical density was 1.0 and prolonging the incubation for 4 h after induction. The cells were then harvested, washed with PBS, and enzymatically lysed (in 50 mm Hepes, pH 7.5 containing 1 mm 2-mercaptoethanol, 150 mm NaCl, 5 mm EDTA, and 1 mm phenylmethylsulfonyl fluoride), and ammonium sulfate was added to the clear homogenate up to 60% saturation. The precipitate was recovered, redissolved in buffer, dialyzed against 50 mm Hepes, pH 7.1 containing 1 mm 2-mercaptoethanol and loaded onto a DEAE-Sepharose Fast Flow column equilibrated with the same buffer. The pass-through was collected, concentrated by ultrafiltration, and fractionated on a Superdex 75 column equilibrated with 20 mm Hepes, 1 mm MgCl_2_, 150 mm NaCl, pH 7.5.

##### Enzyme Assay

The enzymatic activity was routinely measured at 32 °C in 20 mm Hepes, pH 7.5 containing 1 mm MgCl_2_, 150 mm NaCl, 0.25 mm NADP^+^, 0.1 mg/ml BSA in the presence of 0.6 mm Glc-1-P, 0.08 mm Glc-1,6-P_2_, 0.01 mg/ml glucose-6-phosphate dehydrogenase, 0.01 mg/ml phosphoglucose isomerase, and 0.0032 mg/ml phosphomannose isomerase. The activity was followed spectrophotometrically at 340 nm, and the reduction of NADP^+^ to NADPH was recorded. When the effect of calcium ions was tested, the enzymatic activity was measured as described above in the presence of calcium chloride ranging from 0 to 400 μm.

##### Limited Proteolysis

Purified WT, F119L, and R141H were incubated (0.4 mg/ml) with thrombin (500 milliunits/μg of protein) in 20 mm Hepes, pH 7.5, 150 mm NaCl, 1 mm MgCl_2_ at 37 °C. The experiment was contemporarily performed in the presence of 0.5 mm Glc-1,6-P_2_. Aliquots were withdrawn, and residual phosphoglucomutase activity was assayed (except for R141H) over a period of 2 h. Additional aliquots were withdrawn and treated with acetic acid (10% final concentration) for reverse phase liquid chromatography-mass spectrometry (RP-LC-MS) analysis.

RP-LC-MS analyses were carried out on a Q-ToF-Premiere (Waters) equipped with an Alliance binary pump using a Jupiter C_4_ column (5 μm, 300 Å, 50 mm; Phenomenex). The chromatographic runs were carried out using H_2_O, 0.1% trifluoroacetic (buffer A) and acetonitrile, 0.1% trifluoroacetic (buffer B) from 10 to 80% buffer B in 30 min. All mass spectra were acquired from 500 to 2500 *m*/*z* values. Horse heart myoglobin was used for tuning the Q-ToF-Premiere instrument and for mass calibration. All data were acquired and analyzed by MassLynx 4.0, and peptide identifications were carried out with the assistance of Paws software.

Purified WT-PMM2, F119L, and R141H (1.5 μm) were incubated with 1,2-cyclohexanedione (molar excess of 100) in 100 mm sodium borate, pH 8.5 at 37 °C. The experiment was carried out with or without 0.5 mm Glc-1,6-P_2_. Aliquots were withdrawn after 20, 80, 140, and 200 min of reaction and injected onto an RP-LC-electrospray ionization-MS system. RP-LC-electrospray ionization-MS analyses were carried out as described above. Then, all samples were purified by Microcon filters (EMD Millipore) with a 10-kDa cutoff and incubated with trypsin (enzyme:protein ratio, 1:20 (w/w)) for 90 min at 37 °C. Each sample was loaded on a MALDI plate using 4-hydroxycinnamic acid as matrix, and the peptide fingerprint was recorded on a MALDI-Micro (Waters) using enolase mixture as calibrant.

The WT enzyme was also incubated (1.4 mg/ml) at 37 °C with trypsin (protease:enzyme ratio, 1:20) under the same conditions in the presence of 0.5 mm Glc-1,6-P_2_, 0.5 mm Glc-1-P, or 0.5 mm Glc-1-P plus 0.5 mm vanadate (a PMM2 inhibitor that mimics phosphate). Residual phosphoglucomutase activity was assayed over a period of 4 h.

##### Thermal Stability

Heat-induced melting profiles of WT, F119L, and R141H were recorded by thermal shift assay using an iCycler iQ Real Time PCR Detection System (Bio-Rad). The proteins (0.275 mg/ml) were equilibrated in 20 mm Hepes, pH 7.5, 2 mm MgCl_2_, 150 mm NaCl, 1 mm dithiothreitol, 2.4× SYPRO Orange and distributed in 96-well PCR plates, and appropriate ligand solutions (in water) were added (the final volume was 0.025 ml in each well). The plates were sealed with optical quality sealing tape and heated from 25 to 80 °C at 0.5 °C/min. The excitation wavelength was 490 nm, and the emission wavelength was 575 nm. The experiment was performed in the presence of no ligand, 5 mm EDTA, 0.5 mm Glc-1-P, 0.5 mm Glc-1-P plus 0.5 mm vanadate, 0.5 mm vanadate, and 0.5 mm Glc-1,6-P_2_.

##### Miscellaneous Method

The Bradford colorimetric assay was used for protein quantification (19) using the Bio-Rad Protein Assay with bovine serum albumin as standard.

##### Atomic Model Preparation

The Protein Data Bank 2AMY structure had some problems that needed to be fixed before running PELE. The most important aspect was the lack of two Mg^2+^ ions and the 207–222 loop region. We added the Mg^2+^ ions and this loop by superposition to the PMM1 structure, 2FUC. Importantly, PMM1 has the same loop length and the same initial and final loop residues: PMM1 from Phe^215^-Phe^216^ to Asp^232^-Phe^233^ and PMM2 from Phe^206^-Phe^207^ to Asp^223^-Phe^224^. Moreover, superposition of the four initial and final α-carbons, involving residues 206/215, 207/216, 223/232, and 224–233, gave an r.m.s.d. of only 0.25 Å.

Thus, to build our initial model, we copied the backbone of the loop in PMM1 into PMM2 and predicted the side chain positions with Prime (20). The hydrogen bond network of the initial model was then optimized with the Protein Wizard from Maestro at pH 7 (20). Five different initial ligand positions were prepared by placing the ligand randomly in the solvent (with a relative solvent-accessible area of 1.0) and far away from the active site with Mg-ligand distances >20 Å.

##### PELE Simulations

The PELE (17, 18) algorithm is based on a consecutive iteration of three main steps: ligand and protein perturbation, side-chain prediction, and minimization. Ligand perturbation involves a random translation and rotation. Protein perturbation is based on the displacement of α-carbon according to an anisotropic network model. Next, PELE proceeds by optimizing all side chains local to the ligand (within a user-defined distance) together with the “hot” side chains (side chains with the largest energy increase) along the anisotropic network model perturbation. The last procedure involves the minimization of the entire system. These three steps compose a move that is accepted (a new local minimum) or rejected based on a Metropolis criterion; the collection of accepted steps forms a stochastic trajectory. The combination of ligand and protein backbone perturbations results in an effective exploration of the protein energy landscape capable of reproducing large conformational changes associated with ligand migration. PELE uses an all-atom optimized potentials for liquid simulations force field (21) with the Onufriev-Bashford-Case continuum solvent model (22) including a non-polar term approximation (with a 0.15 m ionic strength). Two different exploration runs, a global free search and a local refinement, were performed in this study.

The global search is performed by combining long (6-Å) and short (1.5-Å) ligand perturbation steps with a 75/25% probability, respectively. Rotations were kept in the 0–90° range. Furthermore, a randomly chosen search direction is kept for two Monte Carlo steps, allowing a more complete exploration of the entire protein surface. We should emphasize here that no information of the bound structure is used to drive the search. Anisotropic network model perturbation included the lowest six modes with maximum displacements of the α-carbon of 1 Å. Within the lowest six modes, a randomly chosen mode was kept for six steps to facilitate large conformational exploration.

The local search used translations of 0.5 Å and rotations in the 0–180° range. Furthermore, to keep the ligand in the active site, the random search direction was maintained for one iteration.

For each accepted Monte Carlo step, the binding energy of the ligand was estimated by computing the protein-ligand interaction energy at the given geometry of the protein-ligand complex. Clearly, these energies do not aim to reproduce absolute binding free energies but to identify minima.

##### Structure Analysis

Residue percent accessibility was calculated with PSA v2.0 (23), which uses the rolling probe algorithm (24). We assigned secondary structure with SEGNO (25). Active site residues were identified with DrosteP (26). The figure of superimposed proteins was prepared with Chimera (27).

## RESULTS

### 

#### 

##### Unconstrained Ligand Exploration Produces Two Binding Modes of α-Glucose 1,6-Bisphosphate

The 2AMY structure deposited in the Protein Data Bank represents a good starting point to analyze PMM2 at the atomic level. The enzyme is made up by a core (residues 1–81 and 189–247) and a cap (residues 86–185) domain connected by hinge peptides. Each domain can be nicely superimposed onto the homologous counterparts seen in PMM1 (Protein Data Bank codes 2FUC and 2FUE) (9) and in PMM_LEIME (Protein Data Bank codes 2I54 and 2I55) (10). Clearly, in 2AMY, the domains adopt an open conformation as expected because no sugar ligands were present during crystallization.

In 2AMY, the coordinates of structural Mg^2+^ ions and those of residues 213–215 are missing, the long loop (amino acids 207–222) has relatively high temperature factors, and its orientation is quite different from that found in homologous enzymes. The corresponding loops in PMM1 or PMM_LEIME are well ordered and bind Mg^2+^. Due to the disorder of this loop in 2AMY, Asp^217^ is quite far from the other two residues, Asp^12^ and Asp^14^, that form an acidic triad that binds Mg^2+^ and contributes to catalysis (Asp^19^, Asp^21^, and Asp^226^ in PMM1 and Asp^10^, Asp^12^, and Asp^215^ in PMM_LEIME). For this reason, we generated a native-like initial model grafting the backbone of the loop and the Mg^2+^ ion from PMM1, predicting the side-chain conformations, and minimizing the resulting model (see “Experimental Procedures” for more details and supplemental Structure 1). Additionally, a reference bound complex was obtained by superposition of our initial model to the holo-PMM_LEIME (Protein Data Bank code 2I55) and by copying the glucose 1,6-bisphosphate ligand into our initial PMM2 model. Such a reference compound allowed us to qualitatively assess the ligand evolution along the free migration performed in PELE (17, 18).

**FIGURE 1.**
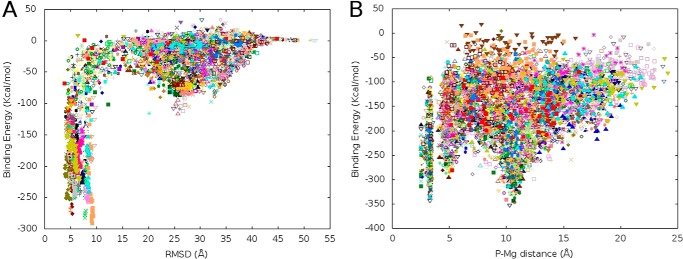
**Ligand binding energy profiles computed by PELE.** *A*, binding energy profile in the full surface exploration against the ligand r.m.s.d. (heavy atom r.m.s.d.) to the bound reference structure. *B*, binding energy profile against the P-Mg distance along the ligand refinement process in the active site. In both panels, each *dot* corresponds to a binding energy (see “Experimental Procedures”) obtained from a protein-ligand complex obtained in PELE sampling. Different colors correspond to processors.

Fig. 1*A* shows the protein-ligand interaction energy against the ligand r.m.s.d. to the bound reference complex along the PELE 200-independent trajectory global search. As mentioned, the bound reference structure serves only as an indication of the active site area and not of the exact position. The results clearly indicate that the ligand finds and discriminates the binding site with all other ligand positions on the surface involving significantly higher interaction energies (a second surface minimum at ∼25–30 Å corresponds to a weak interaction with a second Mg^2+^ ion present in the 221–226 loop). We should emphasize that the ligand is initially placed far from the active site (∼30 Å) and is free to explore with no bias to the binding site. A supplemental movie illustrates some binding trajectories. Interestingly, we found the same number of trajectories where the ligand enters the binding site, interacting with the Mg^2+^ ion, with either of the two phosphate atoms, P or P′. Moreover, the energies associated with these two different binding modes are similar.

To distinguish the possible preferred binding mode, we proceeded by running a ligand refinement search. This refinement step involves small ligand translations and large rotations within the active site, allowing it to reorient but not to move away. Fig. 1*B* shows the protein-ligand interaction energy against the P-Mg distance during the refinement exploration. For further insight into the preferred binding mode, the starting configuration for the 200 refinement trajectories had the same ligand orientation: P′ in contact (∼4 Å) with the Mg^2+^ ion; at this starting conformation, the P-Mg distance is ∼10 Å. After a few PELE Monte Carlo steps, however, the distribution of P-Mg and P′-Mg binding modes reached ∼50%. This is seen in Fig. 1*B* where two equally populated energy minima corresponding to both orientations (P-Mg values of ∼4 and ∼10 Å) are observed. The final model structures, which can be superposed with a protein all-heavy atom r.m.s.d. of 0.6 Å, are provided as supplemental Structures 2 (P-Mg) and 3 (P′-Mg).

**FIGURE 2.**
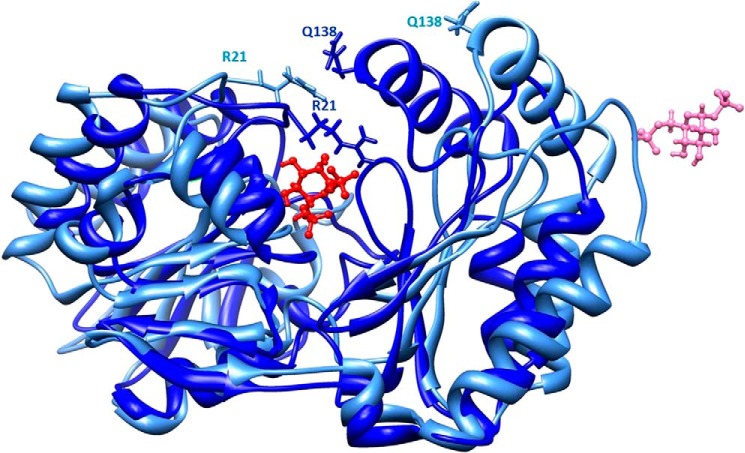
**Protein closure along ligand binding.** The initial model in open conformation is shown in *pale blue*, and the model in closed conformation is shown in *dark blue*. Side chains of two residues that come close upon domain closure, Arg^21^ and Gln^138^, are shown as *sticks*. The initial and final positions of the ligand are shown in *pink* and *red*, respectively, with a *ball and stick* representation. The images were drawn with Chimera (27).

PELE exploration is coupled with large backbone motion, allowing us to monitor closing or opening associated with the ligand dynamics. As mentioned, the initial model obtained from the 2AMY crystal represents an open state. Along with the binding process, however, we clearly observe the closing of both domains around the bound ligand; the supplemental movie clearly shows the closing process. A static view of domain closure is also shown in Fig. 2. The distance between Arg^21^ and Gln^138^ α-carbon experiences the largest change from 20 to 7.5 Å, moving from open to closed conformations, and the exposure of Arg^21^ to solvent changes dramatically passing from 98% in 2AMY to 15% in the closed model. It was interesting to observe that this residue is preceded by a short stretch (^18^TAP^20^) in polyproline II conformation. Although this type of secondary structure is quite rare (28), in all structures solved by x-ray, a polyproline II stretch is observed in the same position. This is true not only for relatively close homologs (Protein Data Bank codes 2FUE, 2FUC, 2I54, and 2I55) but also in more distant ones (Protein Data Bank codes 3F9R and 4BND) (29).

**FIGURE 3.**
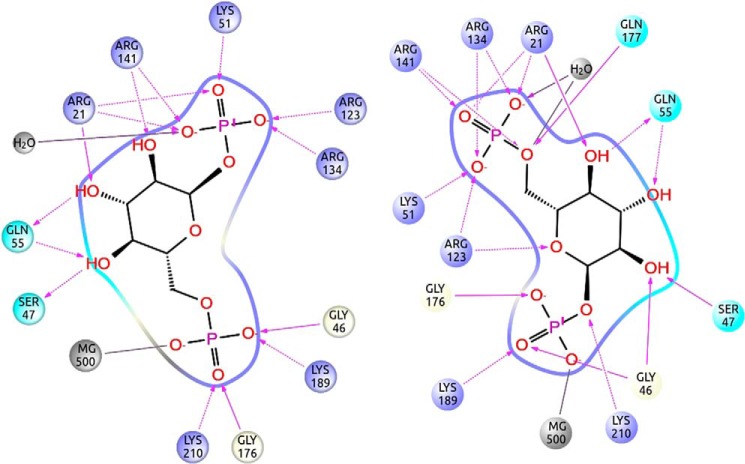
**Ligand binding interactions.** Shown is the active site protein-ligand interaction scheme for the P-Mg and P′-Mg binding modes obtained along the refinement sampling. The structures can be downloaded from the supplemental information.

**FIGURE 4.**
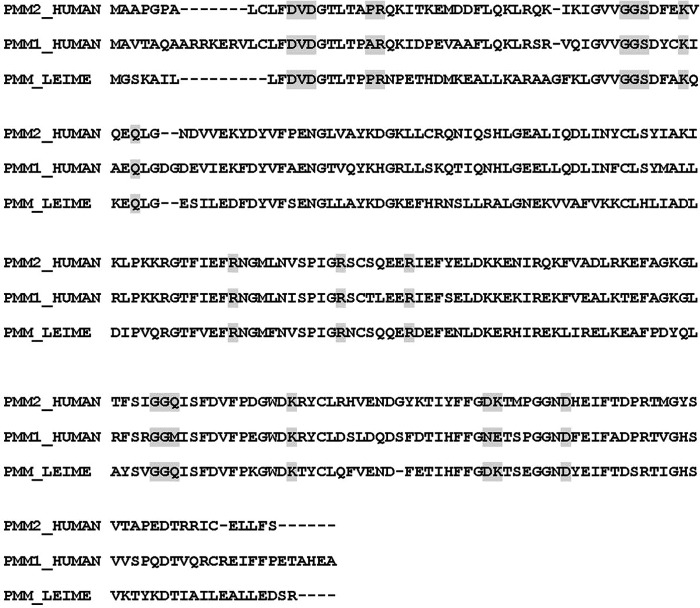
**PMM2, PMM1, and PMM_LEIME sequence alignment.** Sequence alignment of PMM2, PMM1, and PMM_LEIME is shown. Active site residues are *highlighted*. The alignment shows that active site residues are less conserved between the two paralogous human enzymes than between PMM2 and the protozoan enzyme ortholog.

Fig. 3 shows the protein-ligand interaction diagrams corresponding to the two binding modes. The orientations of the ligand in the two models are almost symmetrical and can be interconverted by rotation around an axis passing through O5 (*i.e.* the oxygen in the ring) and C3 (*i.e.* the carbon opposite to it in the ring). As seen in Fig. 3, the interactions between the ligand and the protein are the same in the two orientations provided that we exchange the phosphate atoms. Residues lining the active site pocket can be precisely identified in the closed models, whereas they are ill-defined in 2AMY where domains are far apart. Although amino acid identity between PMM2 and PMM_LEIME is lower (54.8%) than between the two orthologous human enzymes (63.7%), the active site residues are more conserved with respect to PMM_LEIME (see Fig. 4). Active site residues mostly belong to the core domain (residues 1–81 and 189–247). The two domains come closer upon binding and are bridged by the bisphosphate sugar: one phosphate is bound to Lys^189^ of the core domain (Lys^188^ in PMM_LEIME), and the other phosphate is bound to Arg^134^ and Arg^141^ of the cap domain (Arg^133^ and Arg^140^ in PMM_LEIME). A high concentration of positively charged residues is observed in the contact area between domains where Arg^21^ and Arg^141^ (Arg^19^ and Arg^140^ in PMM_LEIME) should act like clasps as seen in Fig. 2.

##### Limited Proteolysis Reveals Conformational Changes Induced by Ligand Binding

**FIGURE 5.**
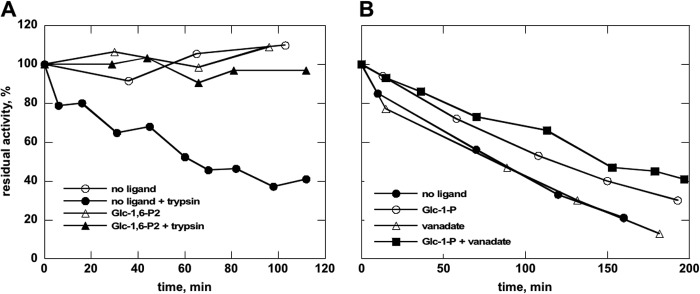
**Limited proteolysis of wild type phosphomannomutase2 with trypsin monitored by assaying residual mutase activity.** Purified wild type phosphomannomutase2 (1.4 mg/ml in 20 mm Hepes, pH 7.5 containing 150 mm NaCl and 1 mm MgCl_2_) was incubated at 37 °C with (or without) trypsin (protease:enzyme ratio, 1:20) in the presence of 0.5 mm Glc-1,6-P_2_ (*A*) or 0.5 mm Glc-1-P, 0.5 mm vanadate, or 0.5 mm Glc-1-P plus 0.5 mm vanadate (*B*). Residual phosphomannomutase activity is reported as a percentage with activity at time 0 defined as 100%. The protective effect of ligands increases as negative charges are added.

Sugar bisphosphate binding makes the structure of PMM2 more compact. This effect was seen *in silico* (Fig. 2) and can be experimentally tested by limited proteolysis by incubating the enzyme with trypsin in the presence or absence of Glc-1,6-P_2_ and measuring residual phosphomannomutase activity (Fig. 5*A*). We observed that binding bisphosphate sugar completely protected the enzyme (Fig. 5*A*, *triangles*). Glc-1P had only a partial protective effect (Fig. 5*B*, *open circles*) that was potentiated by the addition of vanadate, a close structural and chemical mimic of phosphate (Fig. 5*B*, *filled squares*).

To further probe conformational changes in PMM2, we targeted the clasp between domains where the largest displacements are expected using a protease more selective than trypsin. Interestingly, Arg^21^ belongs to a stretch of amino acids, ^11^FDVDGTLTAPRQKITKEMDD^30^, where a Pro is found in position P2, a hydrophobic amino acid is found in position P3, nonacidic amino acids are found in positions P1′ and P2′, and Asp is found in position P10. This stretch represents the only thrombin cleavage site in PMM2. We carried out limited proteolysis on the wild type enzyme as well as on the R141H and F119L mutants, both of which are clinically relevant. Mutation (to His) of Arg^141^ in the active site (see Fig. 3) lowered activity to approximately one-hundredth of that of wild type (data not shown). Phe^119^, on the other side, shifts the dimeric population toward the monomer (11), facilitating the access to proteases. We reasoned that sensitivity to thrombin would be maximal for the monomeric mutant F119L, that Glc-1,6-P_2_ would induce domain closure and hence Arg^21^ protection, and that its effect would be minimal on the inactive mutant R141H.

**FIGURE 6.**
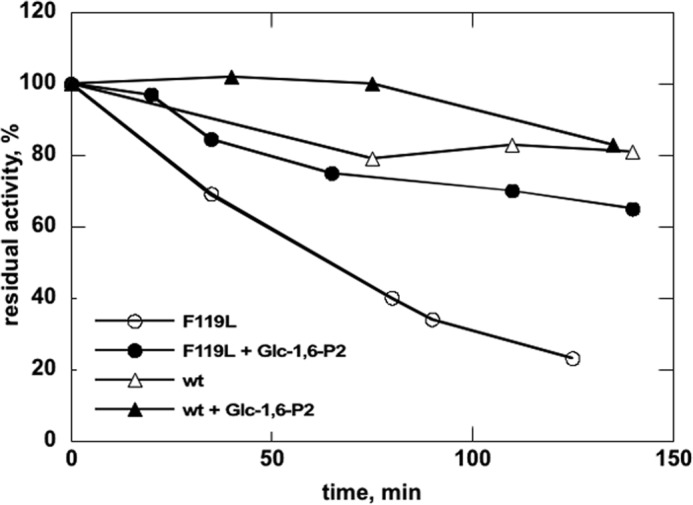
**Wild type and F119L proteolysis with thrombin monitored by assaying residual mutase activity.** Purified wild type or F119L phosphomannomutase2 (0.4 mg/ml in 20 mm Hepes, pH 7.5 containing 150 mm NaCl and 1 mm MgCl_2_) was incubated at 37 °C with thrombin (500 milliunits/μg of protein) with or without 0.5 mm Glc-1,6-P_2_. Residual phosphoglucomutase is reported as a percentage with activity at time 0 defined as 100%. Aliquots were also analyzed by mass spectrometry.

PMM2 wild type or mutants were incubated for different times in the presence of thrombin with or without 0.5 mm Glc-1,6-P_2_. After inactivation of the protease, the residual mutase activity of wild type or F119L was assayed under standard conditions (R141H lacks activity). The results (Fig. 6) clearly show a decrease of activity with incubation time, indicating that Arg^21^ is accessible to the protease. Upon ligand binding, however, PMM2 becomes more resistant to thrombin, an effect significantly more strong in F119L. Kinetic data shown are representative of three independent experiments.

**FIGURE 7.**
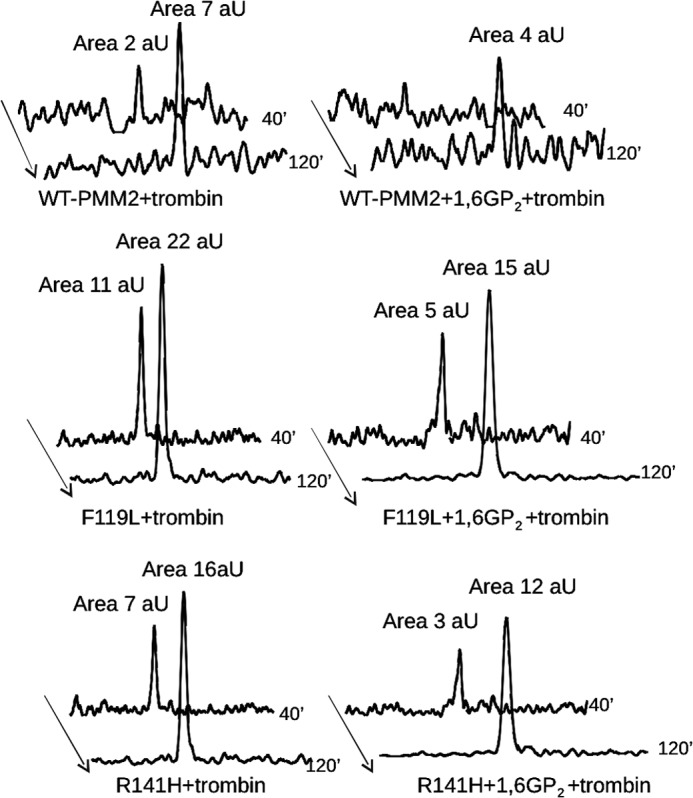
**Limited proteolysis of wild type, F119L, or R141H phosphomannomutase2 with thrombin monitored by mass spectrometry.** The extracted ion chromatograms relative to the formation of peptide 2–21 are reported for wild type (*A* and *B*), F119L (*C* and *D*), and R141H (*E* and *F*) phosphomannomutase2. Samples were incubated in the absence (*A*, *C*, and *E*) or presence (*B*, *D*, and *F*) of α-Glc-1,6-P_2_ (*1,6GP_2_*) with thrombin for 40 or 120 min. Peak areas were calculated by MassLynx 4.0 software and are reported as arbitrary units (*aU*).

**FIGURE 8.**
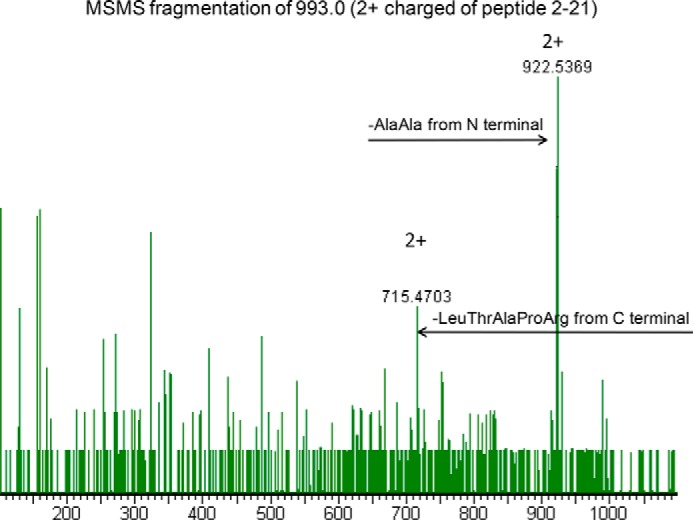
**Identification of the peptide generated by thrombin hydrolysis of phosphomanomutase2.** The electrospray ionization-MS-MS spectrum of the peak at *m*/*z* 993.0, corresponding to the doubly charged ion of 1984.0 Da attributed to peptide 2–21, shows two major fragments that are compatible with the loss of Ala-Ala from the N terminus (doubly charged y-series fragment at 922.53) and with the loss of Leu-Thr-Ala-Pro-Arg from the C terminus (doubly charged b-series fragment at 715.47).

Time course samples at 0, 40, and 120 min were also analyzed by RP-LC-MS analysis (Fig. 7). As expected, all samples were hydrolyzed in two main fragments corresponding to peptides 2–21 (the initial Met is removed in mature PMM2) and 22–245, respectively, confirming that Arg^21^ is the primary thrombin cleavage site. In the absence of ligand, release of peptide 2–21 was faster for F119L, slower for R141H, and even slower for WT (Fig. 7, *A*, *C*, and *E*). Interestingly, the presence of ligand protected against proteolysis of not only the active proteins (Fig. 7, *B* and *D*) but also the R141H inactive mutant (Fig. 7*F*). Peaks in Fig. 7 are extracted from RP-LC-MS chromatograms at a value of *m*/*z* 993.0, corresponding to the doubly charged ions of a species at a monoisotopic mass of 1984.0 Da. This mass value is solely compatible with the fragment 2–21 in the protein sequence digested by trypsin-like proteases. To further confirm the identity of the peptide, the ion at *m*/*z* 993.0 was fragmented by gas collisions in an electrospray ionization-tandem mass spectrometry (MS-MS) experiment. Two major fragments compatible with the loss of Ala-Ala from the N terminus (doubly charged y ion at 922.53) and with the loss of Leu-Thr-Ala-Pro-Arg from the C terminus (doubly charged b ion at 715.47), were recorded (Fig. 8).

**FIGURE 9.**
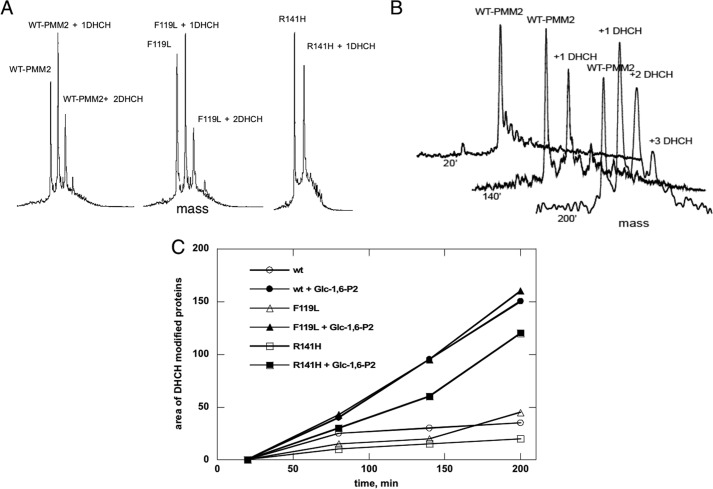
**Chemical modification of reactive arginines in phosphomannomutase2 monitored by mass spectrometry.** *A* shows deconvoluted RP-LC-MS mass spectra of WT-PMM2, F119L, and R141H incubated with 1,2-cyclohexanedione (molar excess of 100) for 140 min, revealing the different modification levels of the three proteins. *B* shows the time course modification of WT-PMM2, revealing unmodified protein (molecular mass of 27,950.25 Da) and mono- and di-modified species (molecular masses of 28,061.50 and 28,173.10 Da). *C* shows the graph of the area of the peaks of deconvoluted spectra relative to 1,2-cyclohexanedione-modified WT-PMM2, F119L, and R141H with or without Glc-1,6-P_2_ measured at different incubation times. ′, minutes.

**FIGURE 10.**
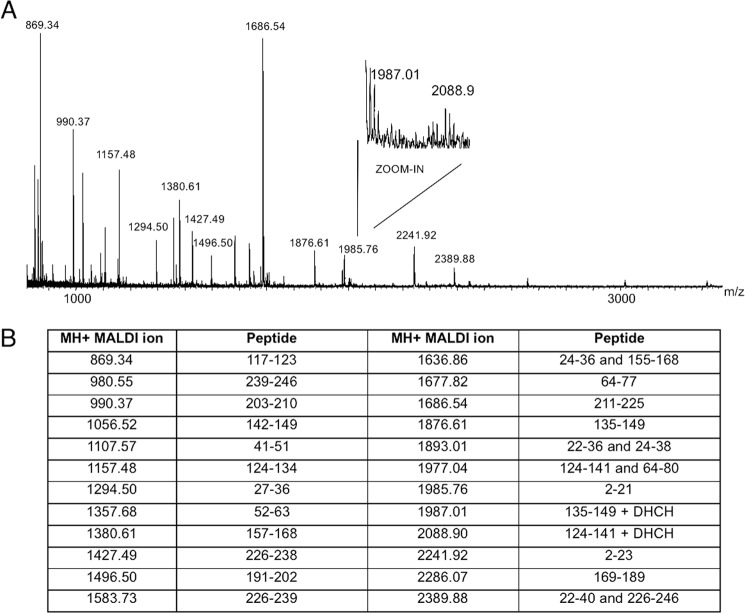
**Identification of reactive arginines in phosphomannomutase2 monitored by MALDI mass spectrometry.** *A* shows the MALDI mass spectrum of WT-PMM2 incubated with 1,2-cyclohexanedione (molar excess of 100) for 140 min and digested by trypsin for 90 min. *B* shows the list of MH^+^ values measured by MALDI MS and attributed to tryptic peptides. Values at 1987.01 and 2088.90 atomic mass units were compatible with peptides 135–149 and 124–141 alternatively modified by 1,2-cyclohexanedione at Arg^141^ and Arg^134^, respectively.

To further confirm the *in silico* conformational changes in the PMM2 active site, we used a specific reagent, 1,2-cyclohexanedione (DHCH), that preferentially modifies reactive arginines (30). This compound is able to reversibly modify the guanidine group, generating a *N*^7^,*N*^8^-(1,2-dihydroxycyclohex-1,2-ylene)-l-arginine (DHCH-arginine) with a protein mass increase of 112 Da for each modified arginine. Purified proteins (1.5 μm) were incubated at 37 °C with DHCH with or without 0.5 mm Glc-1,6-P_2_. Aliquots of incubation mixtures were analyzed by RP-LC-MS after 20, 80, 140, and 200 min of reaction, and mass spectra of each protein, modified or not by DHCH, were recorded (Fig. 9). As reported in *A* and *B*, WT-PMM2 and F119L were progressively modified with up to two DHCH molecules added after 200 min. Conversely, only one DHCH adduct was observed for R141H under the same experimental conditions, demonstrating that Arg^141^ is one of the targets for DHCH. The area of the peaks of deconvoluted spectra relative to the modified forms of WT-PMM2, F119L, and R141H with or without Glc-1,6-P_2_ was measured at different incubation times. The kinetics of DHCH modification was largely reduced (Fig. 9*C*) in presence of the ligand, indicating that target arginines become protected upon binding. The identity of the modified residues, Arg^141^ and Arg^134^, was confirmed by running MALDI spectra of tryptic digestions of DHCH-modified proteins with or without Glc-1,6-P_2_ (Fig. 10, *A* and *B*). Both residues belong to the active site (Fig. 3).

**FIGURE 11.**
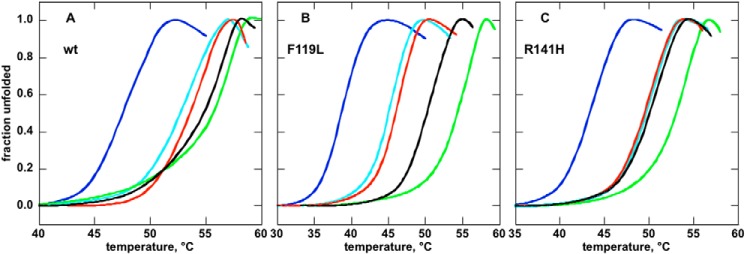
**Thermal stability of wild type, F119L, or R141H phosphomannomutase2 monitored by thermal shift assay.** The profiles (*A*, WT; *B*, F119L; *C*, R141H) were assessed by thermal shift assays. Proteins (0.275 mg/ml in 20 mm Hepes, pH 7.5, 2 mm MgCl_2_, 150 mm NaCl, 1 mm dithiothreitol, 2.4× SYPRO Orange) were heated from 25 to 80 °C at 0.5 °C/min, and fluorescence was recorded (excitation, 490 nm; emission, 575 nm). The experiment was performed in the presence of no ligand (*light blue*), 5 mm EDTA (*blue*), 0.5 mm Glc-1-P (*red*), 0.5 mm Glc-1-P plus 0.5 mm vanadate (*black*), and 0.5 mm Glc-1,6-P_2_ (*green*).

The behavior of R141H was somewhat unexpected. To test its ability to bind Glc-1,6-P_2_, we carried out thermal shift assay. This technique exploits the fact that ligand binding increases the melting temperature of a protein. R141H was indeed stabilized by Glc-1,6-P_2_ (Fig. 11*C*) although to a lesser extent than WT (Fig. 11*A*) or F119L (Fig. 11*B*). We observed that Glc-1-P binding stabilizes WT and F119L. The effect of the monophosphate sugar was potentiated by the addition of vanadate, which in the case of WT reaches the effect of Glc-1,6-P_2._ In contrast, R141H was not stabilized by the sugar monophosphate irrespective of the presence of vanadate.

##### Melting Curves Are Representative of Two Independent Experiments

**FIGURE 12.**
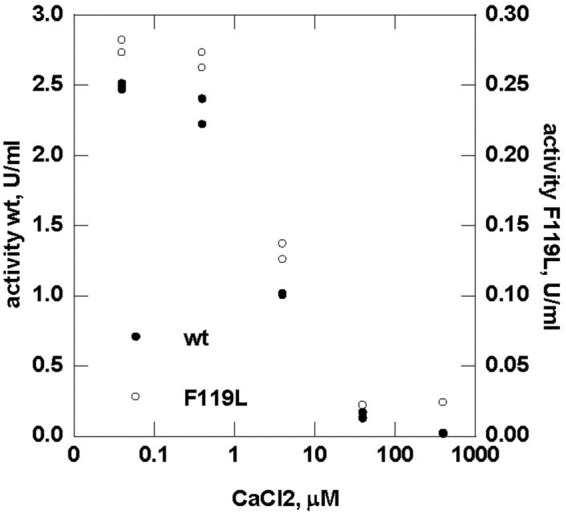
**Effect of calcium ions on the enzymatic activity of wild type and F119L phosphomannomutase2.** Phosphomannomutase activity of wild type and F119L was measured in the presence of calcium chloride ranging from 0 to 400 μm.

Finally, we also tested for the importance of the Mg^2+^ ion. We observed that Mg^2+^ is critical for protein stability (and for activity; data not shown) because curves obtained in the presence of EDTA (Fig. 12, *open circles*) are shifted to lower temperatures compared with those obtained in the presence of the ion (Fig. 12, *open triangles*). Importantly, Mg^2+^ cannot be substituted by other divalent ions, but on the contrary, Ca^2+^ acts as an inhibitor (Fig. 12). These findings reinforce the idea that 2AMY, which lacks a proper description of the ion binding site and the bound Mg^2+^ ion, cannot represent the native-like structure for PMM2.

## DISCUSSION

Patients suffering from PMM2_CDG have residual enzymatic activity, and their parents, who have only one mutated allele and 50% activity, are asymptomatic. Drugs such as pharmacological chaperones and/or activators (31) providing an increase in activity would be beneficial for them. The scarcity of biochemical and structural data, however, slows down the search for drugs. Our hypothesis states that the current crystallographic structure of PMM2 cannot be used for rational drug discovery because domains are too far apart, and Mg^2+^ ions are missing. Extensive computational and experimental studies have confirmed this point. Using recently developed computational techniques modeling non-biased ligand migration, active site search, and binding, we observed spontaneous binding in the same region as observed in homologous enzymes (PMM_LEIME). The binding process was coupled with significant protein reorganization in which a closed structure that required large domain motion was adopted.

Parallel to the all-atom modeling, limited proteolysis with trypsin, site-directed proteolysis with thrombin, and selective chemical modification confirmed closing of the enzyme upon ligand binding. Activity assays and thermal shift assays (this study and Ref. 11) showed the necessity of adding Mg^2+^ (and thus the missing loop interacting with it) for enzyme function and stability. Thus, overall, the PMM2 crystal structure represents a very poor model for rational drug design even for current flexible (receptor) docking techniques.

Our study indicates that Glc-1,6-P_2_ can bind in two different modes. Because of the large symmetry of this sugar ligand, we found two equivalent bound structures where the P or P′ phosphates bind to Mg^2+^. The presence of these two phosphate groups together with their large negative charge is crucial for domain closure. Corroborating experimental evidence comes from limited proteolysis and thermal shift assays. In fact, the resistance of the protein is minimal with a monophosphate sugar; intermediate with a monophosphate sugar plus vanadate, an inhibitor that mimics phosphate and recreates a complex similar to sugar 1,6-bisphosphate in the active site; and maximal with bisphosphate sugar.

Our models offer insights about the enzyme mechanism and identify active site residues. This is important to address therapy because mutations affecting these residues cannot be rescued with pharmacological chaperones (32). In addition, it is also important for diagnoses. As already mentioned, most patients are compound heterozygotes carrying one mutation, R141H (6). For these patients, we expect a residual activity below 50% and above 0%. What can we expect for people carrying two missense mutations but not R141H? Most mutations, which are pathological in association with R141H, might not be pathological by themselves (*i.e.* A/R141H and B/R141H are pathological, and A/B not). We made a survey of the data available in the literature excluding the case where a stop codon, a frameshift, or alternative splicing was observed. We found that approximately 40% of the patients who do not carry R141H have a mutation that affects the active site as defined in this study (Figs. 3 and 4). This effect was reported for K51R, R123G, R123Q, G175R, and G176V (5, 33–35). We can expect that a mutation affecting these residues reduces functionality to such an extent that when combined with a second hypomorphic mutation will result in less than 50% residual activity.

Summing up these observations, we put forward the hypothesis that the occurrence of one mutation in the active site represents a condition sufficient but not necessary for pathology. It is worth remembering that other mutations such as F119L and D65Y not occurring in the active site are deleterious enough to confer pathology in the absence of R141H.

In the course of the experiments designed to validate the models generated by PELE, we learned that the common mutant R141H, which is inactive, binds bisphosphate sugar and undergoes domain closure. As seen in Fig. 3, numerous positive residues interact with the outer phosphate (away from the Mg^2+^ ion) and stabilize domain closure. Thus, the influence of this mutation on enzymatic activity does not seem to be related to sugar binding. Awareness of the functioning of R141H is important when studying heterodimers with active mutants because the vast majority of PMM2-CDG patients have this type of enzyme.

In summary, combining modern computational techniques capable of modeling non-biased ligand migration and protein dynamics with limited and site-directed proteolysis, we have produced the first accurate model of a ligand-bound complex for PMM2. Moreover, our studies provide valuable insight into the behavior of key mutants.

## Supplementary Material

Supplemental Data

Supplemental Data

## Footnotes

4 The abbreviations used are:

PMM: phosphomannomutase
PELE: Protein Energy Landscape Exploration
Glc-1,6-P_2_: α-glucose 1,6-bisphosphate
Glc-1-P: glucose 1-phosphate
CDG: congenital disorder of glycosylation
RP: reverse phase
r.m.s.d.: root mean square deviation
DHCH: 1,2-cyclohexanedione
PMM_LEIME: phosphomannomutase from *L. mexicana*.
