# Supplementary material for: Conformational Response to Ligand Binding in Phosphomannomutase2: INSIGHTS INTO INBORN GLYCOSYLATION DISORDER
Source: J Biol Chem. 2014 Oct 16;289(50):34900–10. doi: 10.1074/jbc.M114.586362 (PMC4263888; doi:10.1074/jbc.M114.586362)
Supplement: Supplemental Data [file supp_289_50_34900__index.html]

Conformational response to ligand binding in phosphomannomutase2, insights into inborn glycosylation disorder — Conformational Response to Ligand Binding in Phosphomannomutase2 — Modeling the Conformational Transition in PMM2 — Supplemental Data 

# Conformational Response to Ligand Binding in Phosphomannomutase2

## Supplemental Data

**Files in this Data Supplement:**

- Supplemental Movie (.mpg, 3.3 MB) - A movie compiling three different global search trajectories
