# Supplementary material for: Conformational Response to Ligand Binding in Phosphomannomutase2: INSIGHTS INTO INBORN GLYCOSYLATION DISORDER
Source: J Biol Chem. 2014 Oct 16;289(50):34900–10. doi: 10.1074/jbc.M114.586362 (PMC4263888; doi:10.1074/jbc.M114.586362)
Supplement: Supplemental Data [file supp_289_50_34900_v2_index.html]

Conformational Response to Ligand Binding in Phosphomannomutase2 — Modeling the Conformational Transition in PMM2 — Supplemental Data 

# Conformational Response to Ligand Binding in Phosphomannomutase2

## Supplemental Data

**Files in this Data Supplement:**

- Supplemental Structure 1 (.pdb, 357 KB) - Initial model for ligand migration search with corrected Mg ions and loop
- Supplemental Structure 2 (.pdb, 322 KB) - P-Mg binding mode structure
- Supplemental Structure 3 (.pdb, 315 KB) - P'-Mg binding mode structure
